# ‘Don’t Put the Cart before the Mule!’ Challenging Assumptions Regarding Health-Related Treatment Practices of Working Equid Owners in Northern India

**DOI:** 10.3390/ani11051307

**Published:** 2021-05-01

**Authors:** Caroline Nye, Tamlin Watson, Laura M. Kubasiewicz, Zoe Raw, Faith Burden

**Affiliations:** 1 Centre for Rural Policy Research, The University of Exeter, Devon EX4 4PJ, UK; 2 The Donkey Sanctuary, Sidmouth EX10 0NU, UK; tamlin.watson@thedonkeysanctuary.org.uk (T.W.); laura.kubasiewicz@thedonkeysanctuary.org.uk (L.M.K.); zoe.raw@thedonkeysanctuary.org.uk (Z.R.); faith.burden@thedonkeysanctuary.org.uk (F.B.)

**Keywords:** working equids, theory of planned behaviour, donkeys, mules, decision-making, health, welfare, husbandry, ethnoveterinary

## Abstract

**Simple Summary:**

Donkeys and mules are still commonly worked across the world in place of mechanical vehicles. They are prone to various diseases and welfare issues, often as a result of poor working conditions. A proportion of the associated literature and a number of interventions are still driven by the assumption that the owners of working equids base their decisions on how to treat their animal upon economic factors or access to veterinary services. This paper uses a mixture of qualitative and quantitative methods to understand the reasons behind why equid owners choose to treat their animals in the way that they do. It explores the various means by which they choose to do this and the avenues available to them. It identifies four factors which influence how and why owners of working equids choose to treat their animals in a particular way and highlights in particular the frequency with which traditional methods or ‘home remedies’ play a significant role in attempts to remedy the poor health of an animal. The findings demonstrate a greater need to validate traditional methods used, in order to promote better health and to reduce risk, as well as the requirement to tailor animal welfare interventions according to these influencing factors to encourage more sustainable outcomes for both animals and owners.

**Abstract:**

This paper challenges assumptions that the health management of working equids among some of India’s poorest communities is mainly dependent upon income, economic influence, or access to veterinary services. Using a mixed-methods approach, hierarchies of treatment practices are revealed through an examination of the ‘lived experience’ of equid owners in brick kilns and construction sites in northern India. Semi-structured interviews with 37 equid owners and corresponding livelihood surveys, combined with data from two focus groups with professional animal health practitioners and the welfare data of 63 working equids collected using the Equid Assessment, Research, and Scoping (EARS) tool, contributed to the findings of the study. Four principal influencing factors were found to affect the decision-making practices of equid owners. Infrastructural factors, community characteristics and experience, owners’ characteristics and experience, and economic factors all impact the belief structures of equid owners. However, without verifying the validity of the treatment measures being employed, some animals are at risk from hazardous treatment behaviours. By understanding decision-making using the theory of planned behaviour, the findings of this study can provide a crucial contribution to informing future interventions involved in the health management and welfare of working equids.

## 1. Introduction

Approximately 600 million people rely upon equids for their livelihoods globally [1], with over 100 million equids estimated to still be worked worldwide [2]. For many of these people, it is often the case that the majority of their income derives from the work carried out with their animals [3,4]. Despite this, the welfare of working equids is known to be among the poorest of any domesticated large mammal anywhere in the world [3]. A working equid’s potential risk of injury, illness, or disease is high, particularly among mules and donkeys [5]. This risk is accentuated by poor welfare management, limited food and water, and extreme working conditions, which some link to the fact that the majority of working equids belong to individuals or families coming from some of the poorest communities [6,7]. While the term ‘working equid’ can refer to horses, mules, and donkeys, it is working donkeys and mules which are more commonly used in less economically developed countries. Numerous studies exist which examine the efficacy of welfare assessment methods [5,8], yet little work has been published which examines the decision-making processes, help-seeking behaviours, or hierarchies of treatment practices belonging to owners of working equids, whether such practices are diagnostic, preventive, palliative, or curative. A variety of treatment options are usually available to equid owners in India, such as the intervention of a veterinary practitioner, seeking the advice of a pharmacist, or choosing to self-treat one’s own animal using home remedies or other traditional methods. The availability of treatments is, however, specific to regions or local contexts. Traditional healthcare remains prevalent in many low- and middle-income countries (LMICs), including India, despite an increase in the availability of allopathic medicine. While allopathic medicine might be prohibitively expensive, or inaccessible to some animal owners, this is not always the case; yet the assumption persists that treatment behaviours are dependent upon economic variables and that the common default to home remedies and self-treatment is primarily a result of economic influence [7,9] or lack of access to veterinary services [10]. Upjohn et al. [10] discovered, for example, that out of six NGO interventions for working equids in the overseas health and welfare sector, the majority involved the provision of veterinary, or veterinary-supervised, services.

This study sought to identify the hierarchies of treatment choice adopted by owners of working equids in northern India, to explore the factors influencing decision-making processes, and to determine the effect of preferred practices on equid welfare using the Equid Assessment, Research, and Scoping (EARS) tool [8,11]. The study was informed by the theory of planned behaviour [12] in order to better understand equid owners’ decision-making and treatment practices. It is critical to understand how owners of working equids make decisions about how to treat their animals for myriad reasons. The primary motive is to promote positive patient outcomes for the animals and, consequently, ensure sustainability of livelihood for owners. Secondly, treatment decisions can have implications for wider global health issues, such as antimicrobial resistance [13,14]. Thirdly, such knowledge can inform future intervention strategies to ensure sustainability of practice. Subsequent insights resulting from this research will be useful for organisational intervention design or campaigns to encourage more sustainable ‘on-the-ground’ treatment decisions regarding the health of working equids globally.

### 1.1. Traditional Remedies and the Decision to Self-Treat

As early as 1998, it was identified that, in less economically developed countries, ‘veterinary services have almost universally been modelled on those of donor countries, with little consideration given to the host country’s particular cultural, economic, and other conditions’ [15] (p. 146). Subsequently, interventions have, for too long, been developed by equid welfare organisations working as ‘silos’ [16] (p. 11), failing to communicate or collaborate towards common goals and building strategies upon assumptions rather than empirical fact.

Traditional methods have long been regarded as ‘quackery’, associated with fraudulent misrepresentations of a practitioner’s ability to diagnose or treat a disease, or as having either zero or negative impact upon treatment recipients [17] (p. 408). However, recognition that ‘communal knowledge and innovation are an integral part of the day-to-day healing and management practices’ [9] (p. 121) of animals in developing countries has led to some projects not only validating local ethnoveterinary practices but also encouraging their continued use. This ensures that owners who might not have access to allopathic veterinary services are sufficiently informed, enabling them to treat their animals effectively based on locally available resources. The Anthra project in Andhra Pradesh and Maharashtra states, India, is one such example [9]. A project belonging to an organisation of female veterinarians, the Anthra project discovered that many of the local practices to treat livestock were effective. They sought to validate various ethnoveterinary treatments in order to develop ‘a protocol that would enable the integration and synthesis of the best and most useful elements of the indigenous and modern systems’ [9] (p. 122). The World Health Organisation itself recognizes the potential contribution of traditional medicine to healthcare and encourages the integration of safe and effective traditional medicine into health service delivery, particularly for poor populations [18].

An ‘overwhelming majority’ of animal owners in less economically developed countries are reliant upon traditional medicine in the treatment of their animals [19] (p. 241). Erosion of use has occurred to some extent due to a progression of allopathic practice, but the prevalence of traditional medicine remains high [20]. Muhammad et al. [19] attribute such a continuation to ‘cost, inaccessibility and other problems associated with western-style healthcare’ [19] (p. 241). The World Health Organisation (WHO), however, recognises that, although accessibility and affordability act as drivers in traditional medicine use, traditional medicine remains popular ‘because it is firmly embedded within wider belief systems’ of the culture of which it is a part [21] (p. 2).

Traditional medicine, where it is not incorporated into the national healthcare system, is often regarded as ‘complementary’ or non-conventional. However, in Africa, almost 80 per cent of the population practice traditional medicine, China alone has 40 per cent still reliant upon it, and many countries in Latin America also continue its use at comparatively high levels [21]. Due to the deep level at which Ayurvedic, yoga, Unani, Siddha, and homeopathic medicine (AYUSH) has become embedded within the Indian medicinal system, India has its own national policy on traditional medicine, has a traditional medicine department within its ministry of health, and regulates both traditional medicine and herbal products [21,22]. According to an earlier WHO report, the Indian government stated that, ‘for 65 per cent of its population, traditional medicine is the only available source of healthcare’ [21] (p. 13). However, even the term ‘traditional medicine’ is complex in India, as it might include rudimentary home-remedies or complex, shop-bought Ayurvedic preparations. The term Ayurvedic refers to the natural system of medicine that has been prevalent in India for several thousand years. A traditional method of treatment, in the context of this paper, includes any non-allopathic remedy which uses herbal medicine (including animal parts or minerals) and/or non-medication therapy, such as massage.

Watson et al. [16] determined that ‘blind spots’ often prevent the sustainability of intervention methods and that, instead, ‘facilitating owners to problem-solve their own solutions would give people ownership and empowerment to succeed’ [16] (p. 11). While a number of studies have been carried out examining equid welfare, few have directly examined causative agents of welfare, such as cultural influence [16] or treatment decision-making practices. Bypassing such information in intervention design is likely to be to the detriment of the animal, the owner, and the efficacy of the intervention itself.

Colic, trypanosomiasis (Surra), lameness, respiratory tract infections, and abscess/wounds/injuries are reported as the five most common equid ailments in India, as identified by equid veterinary practitioners [7]. While regional differences exist across the country, and bearing in mind that biases may exist in these results as cases are significantly underreported, it is likely that such ailments are those for which treatment decisions need to be frequently made by animal owners. However, recognition that ‘only a fraction of sick animals are brought for treatment to veterinarians’ [7] (p. 148) begs the questions of who is treating the rest of these animals, how they are being treated, and what the influences are on the decisions regarding treatment.

### 1.2. The Decision-Making Process

Rather than assess attitudes as predictors of specified behaviours, Ajzen’s [12] theory of planned behaviour (TPB) loosely informed the focus of this study; loosely, as it is impossible to assume that treatment behaviours of equid owners can be entirely volitional, a prerequisite of this theory, as time, (lack of) money, limited power, and the cooperation of others might also impact behaviour. According to Bowden et al. [23], an animal owner’s ‘opinion of the importance of a disease will depend both on how common it is, and the impact it has’ [23] (p. 2). If those owners also have ‘limited access to resources to inform decision-making’ [23] (p. 3) then their ability to make important decisions about managing the health of their animal might be further affected by pressures such as time, finances, or emotions.

However, the role of beliefs is considered sufficiently likely to explain an owner’s treatment behaviour, which, according to the TPB, results from the ‘intention’ to perform a behaviour led by three kinds of beliefs. Behavioural beliefs are those which tie in with perceived negative or positive consequences of an action and the related negative or positive memory associated with a consequence. Secondly, *normative beliefs*, related to perceived social pressures and whether important individuals within a community might approve or disapprove of an action, might also affect behaviour. Finally, *control beliefs* refer to the presence or absence of opportunities and resources available, which affect how an individual perceives their level of control over their behaviour. If all of the above prove favourable, then ‘the more likely it is that a person will form an intention to perform the behaviour in question’ [24]. Feedback loops are believed to reinforce such behaviours and, as a result, cause them to become entrenched societal or cultural norms.

The process of making a decision regarding treating the ailment of a sick animal is, according to the TPB, extremely unlikely to occur spontaneously, but this does not imply that behaviours cannot be flexible or converted into new behaviours. Due to the nature of the process about which a decision is being made—in this case, health—the decision-making process also needs to be considered in terms of how decisions can affect the spread of a particular belief or practice regarding healthcare. Ayalew and Mulatu [25] have discussed how ‘farmers’ indigenous innovations emerge from, and are part of, the traditional farmers’ practices in dealing with routine animal husbandry problems’ [25] (p. 62). They determined that such problems cannot be considered externally to the local culture, biophysical environment, or local networks. To the same end, Tanaka et al. [26] discovered that it is unsafe to assume that only efficacious traditional remedies are repeated and thus spread. The reason for this is that ‘superstitious treatments and maladaptive practices can spread because their very ineffectiveness results in sick individuals demonstrating the practice for longer than efficacious treatments, leading to more salient demonstration and more converts’ [26] (p. 4). They conclude that uptake of a healthcare practice can often be based on limited circumstantial evidence and is instead guided by preconceptions influenced by culture and perceived efficacy, which is not actually reflective of the efficacy of a treatment itself. Adoption of a treatment in some regions, according to Tanaka et al., ‘is potentially a function of the rate of practice demonstration’ [26] (p. 7). Consequently, it is important to identify not only the hierarchies of choice occurring among animal owners, or health treatment providers, but also to examine whether traditional remedies commonly used are detrimental to the well-being of the animals [9]. Fully exploring treatments or identifying the validity of the ethnoveterinary methods described is beyond the scope of this paper. Instead, this paper examines the decision-making process behind the usage of such treatments when compared with other available medical service options for the animals.

## 2. Materials and Methods 

**Figure 1:**
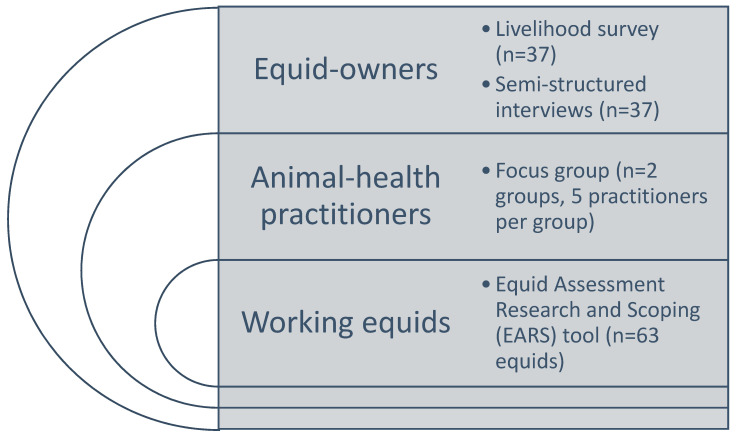
Study methodology.

This study examined the decision-making processes and health treatment choices of equid owners in northern India using a mixed-methods approach, but it was principally based around semi-structured, qualitative interviews (Figure 1). Prior to each interview, a short livelihood survey (Supplementary materials) was carried out with each respondent in order to gather basic data regarding both the individual and their animal/s. In total, two focus groups were held with animal health practitioners, with one each in two of the three regions of study. The methodology also utilised the EARS tool [8] to determine whether any indications existed that suggest that variations in health-treatment choices are associated with different welfare outcomes in working equids. This paper emerged from a larger study which examined the role of veterinary drug retail outlets (locally known as medical stores) on equid welfare [14].

Northern India was selected as a fieldwork site due to the prevalence of working equids in the region, where donkeys and mules are commonly used for work in both brick kilns and on construction sites. Fieldwork was carried out in the months of February and March 2019 in the following three regions of northern India: Faridabad, Haryana; Neemrana, Rajasthan; and Lucknow, Uttar Pradesh. Regions of study included rural and peri-urban areas. Key informants known to have worked for a number of different non-governmental organisations (NGOs) assisted researchers in accessing the communities, and two Indian national research assistants were employed to assist with interpretation. All interviews and EARS assessments were carried out in the domestic areas of work sites when respondents were not working and had rested post-work. These sites consisted of temporary shanty communities, impermanent due to the seasonality of the work and the movement of kilns from place to place according to the availability of resources. 

### 2.1. Respondent Recruitment

A total of 14 brick kilns and 2 construction sites were visited for the purpose of recruiting participants, with a single visit to each site. There was no time limit as to the fieldwork visit but researchers attempted to recruit at least three owners at each site. Study sites known for their high populations of working equids were identified by informants and respondents were recruited on arrival and in situ, with the inclusion criteria being that respondents were responsible for an equid and over 18 years old. Participation was voluntary and unpaid, with consent being obtained verbally and audio-recorded. Two focus groups were also conducted with animal health practitioners working in Faridabad and Neemrana. These participants consisted of both government and NGO-funded veterinarians (one retired) and community animal-health workers (CAHWs). Respondents were recruited using purposive sampling due to the limited availability of such practitioners in both regions.

Small sample sizes for the qualitative methods allowed for a ‘narrow and deep’ analysis of the drivers of treatment behaviours, as opposed to a ‘broad and shallow’ method. This allowed for unexpected themes to emerge inductively through long interviews and encouraged a grounded theory approach [27] whereby a theoretical framework could develop from the empirical data. 

### 2.2. Livelihood Surveys

Livelihood surveys were conducted using digital tablets and a pre-designed Open Data Kit (ODK) Collect software form using pre-set questions (Supplementary materials), and were performed in Hindi by the research assistants in order to save time. Questions collected basic data regarding age, education, gender, and animal-owning behaviours (such as numbers of animals owned and for how long), and the process was kept as simple as possible in order to avoid bias or difficulty on the part of the research assistants. The data were uploaded to a central server in the UK for the purpose of quantitative analysis.

### 2.3. Interview Procedure

Interviews were conducted by one of two principal researchers in English and audio-recorded, with research assistants interpreting into and from Hindi in situ. Recordings were transcribed post-fieldwork. A semi-structured design allowed for new topics to emerge and augment predetermined themes around questions regarding animal welfare, illness, and treatment. Interviews were held both in brick kilns and on construction sites where donkeys and mules were used for the transportation of goods. All locations were rural or semi-rural, with only one site located on the edge of a town.

### 2.4. Focus Group Procedure

Two focus group discussions (Figure 1) were held, with each discussion lasting approximately 65 min. Each focus group was made up of five animal health practitioners. The first focus group was held with practitioners in Faridabad and the second group with practitioners in Neemrana. Open-ended questions were asked regarding the respondents’ knowledge, experience, and perceptions of the treatment practices and decision-making processes of owners of working equids in the chosen study regions. Group discussions were conducted in English, translated in situ (although a minority of respondents were able to reply in English), audio-recorded, and transcribed post-fieldwork.

### 2.5. Welfare Data Procedure

Welfare data were collected by a specialist trained assessor on the research team using the EARS tool. This tool is a resource which incorporates ‘pre-existing validated welfare assessment methods alongside new welfare indicators’ in order to offer the most comprehensive set of welfare indicators to date [8] (p. 1). The welfare of equids owned by interview participants was measured using the tool, with the equids selected according to their proximity to the fieldwork site at the time of interview and the ensured safety of both equid and assessor. Although every attempt was made to assess equids belonging to all owners for all welfare indicators, it was not possible in some cases. Sample sizes for individual questions may, therefore, differ from the total cohort and are provided for individual questions where appropriate. ODK Collect was again used for this purpose, with a separately designed form. All inputted data were uploaded to a central server for analysis purposes.

### 2.6. Data Analysis

#### 2.6.1. Qualitative

Qualitative data were, once transcribed, uploaded to and analysed using Nvivo (V.12.2, QSR International, Pty Ltd, Melbourne, Australia), a qualitative software package. Researchers employed deductive and inductive coding frameworks in order to identify themes from the data. This involved identifying segments of text from transcripts and assigning them codes, which subsequently emerged as themes. Codes could be pre-determined, heuristic, or, as is commonly the case in qualitative analysis, a combination of the two.

#### 2.6.2. Quantitative

Livelihood survey data were cleaned and then uploaded and analysed using IBM SPSS Statistics 26, R (V 3.6.1) [28], and R.Studio (V 1.2.5001) [29].

To test whether equid health differed between types of ‘primary treatment choice’, we used an independent Welch’s t-test. Normal distribution was confirmed using a quantile–quantile (qq) plot for each treatment choice, where deviations from the straight line were minimal. Primary treatment choices were grouped into ‘self-treatment’ and ‘other’, where ‘other’ included the following treatment options: pansari (seller of herbs), medical store, veterinarian (private or government), and NGO veterinarian. This grouping was used due to the small sample sizes of all the treatment types except self-treatment and to reflect our primary interest in the efficacy of self-treatment. Treatment options that included the phrase ‘depends on’ were excluded from the comparison due to uncertainty. To represent the scope of equid health, we followed step one of the Welfare Aggregation and Guidance (WAG) tool outlined in [11]. Following this protocol, four questions from the EARS welfare assessment, namely signs of illness, coat health, respiratory health, and skin system alterations, were aggregated using a decision tree, leading to a ‘health’ score ranging from 0 (worst welfare) to 100 (best welfare) for each animal. These four questions were understood to be the most indicative of welfare in the context of this study. Mean scores for each equid owner were then calculated. 

### 2.7. Ethics

The study was conducted in accordance with the Declaration of Helsinki [30] and the protocol approved by the Executive Board of the Donkey Sanctuary UK, with Project Number 2018-AIM2-TASK4. Informed consent was obtained verbally from all interview and focus group participants, and audio-recorded, prior to participation. Neither the names of individuals nor of work sites were collected or stored post-fieldwork. All data were kept anonymous. Equids were assessed using non-invasive techniques.

## 3. Results

### 3.1. Owner Characteristics

A total of 37 working equid owners were interviewed, with 24 per cent (*n* = 9) in the 18–30 age group, 62 per cent (*n* = 23) in the 30–50 age group, and 14 per cent (*n* = 5) of respondents being over 50. Only five per cent (*n* = 2) of respondents were female. The majority owned only one equid, although four respondents owned between 10 and 21 equids per person.

Education levels among respondents varied, with 54 per cent (*n* = 20) reporting having received no formal education, 24 per cent having completed primary education (*n* = 9), and only 22 per cent (*n* = 8) completing secondary education.

### 3.2. Equid Data

A total of 63 equids, belonging to 32 owners, were assessed, 35 per cent (*n* = 22) of which were donkeys, 57 per cent (*n* = 36) mules, and eight per cent (*n* = 5) horses. The five horses were owned by different people, all of whom also owned mules or donkeys.

Overall, according to the EARS assessment, only 25 per cent (*n* = 16) of the equids assessed were reported as having a general health status of ‘good’. Forty-nine per cent (*n* = 31) presented as ‘fair’ while 25 per cent (*n* = 16) presented as having a ‘poor’ general health status.

According to the owners, the most common health issues, as presented by their animals and in order of frequency mentioned, were colic, eye discharge, constipation, abdominal issues, leg swellings or injuries (including broken legs), lameness, fever, coughs, cold, fatigue, equid trypanosomosis/Surra, tetanus, general wounds, eye wounds, diarrhoea, nasal discharge, abscesses, loss of appetite, respiratory issues, and general injuries. 

Data outlining mortality rates and causes of death were difficult to quantify as few cases of deaths were reported outside of NGO intervention site areas. Most owners who commented reported burying their animal in the ground with salt when an animal died with no report of death. Similarly, few records were kept by owners, and even some members of the professional healthcare sector, concerning incidents of animal disease.

### 3.3. Knowledge of Owners

Eighty-four per cent of respondents (*n* = 31) reported having learned how to look after and treat their animal from family members. Of those remaining, the sources of learning cited were a contractor, a doctor, a guru, and the local village community. One respondent married into a donkey-owning family and had no knowledge of equids prior to that.

Levels of confidence as to their knowledge regarding treating equids for illness ranged from extremely confident to not very confident at all, with fewer expressing a feeling of ‘not very confident’ than ‘somewhat confident’, ‘confident’, or ‘very confident’.

The following section explores the preferred treatment practices of working equid owners in the study regions, as well as identifying those factors which affect the decision-making process regarding treatment choice. While explanation was employed where deemed necessary, it is important to bear in mind that the terms ‘traditional medicine’, ‘home remedies’, and ‘Ayurvedic medicine’ proved ambiguous and were occasionally misunderstood by some of the respondents, as a minority did not know the difference between Ayurvedic and homeopathic medicine or, in some cases, allopathic medicine (often described as English medicine). The term ‘doctor’ also appeared to be ambiguous. For some respondents, ‘doctor’ might have referred to a trained veterinary practitioner, but in some cases the *pansari* was also termed a doctor, requiring further probes by researchers to determine which treatment service was actually being referred to.

### 3.4. Treatment Practices 

The findings demonstrate that when a working equid shows signs of illness, owners in northern India appear to have six available options for healthcare for their animals: (i) self-treat using home remedies; (ii) visit a pansari (retailers of herbs, spices, Ayurvedic medicines, and blends); (iii) seek advice from a government veterinarian (free consultation); (iv) contact a private veterinarian (consultation by fee); (v) refer to a veterinarian working for a non-governmental organisation (NGO); or (vi) go directly to a medical store (pharmaceutical outlet). In some cases, self-treatment might include the decision to do nothing at all, such as continuing to work if the animal suffers an injury or making the decision to reduce an animal’s workload only slightly. It was reported by some respondents that their animals were healthy and suffered few injuries or illnesses, with several respondents claiming that their animal had never required treatment at any time. Of eight respondents reporting that their animal never had any health issues or injuries, six animals presented problems with eye discharge, two with loose stools, one was lame (but still moving), three animals had open wounds, and all had scars on at least one part of their body during the EARS assessment. This suggests that knowledge and perceptions regarding signs of illness or injury were limited among this cohort or that conditions were not considered sufficiently serious to be considered a ‘problem’.

**Figure 2:**
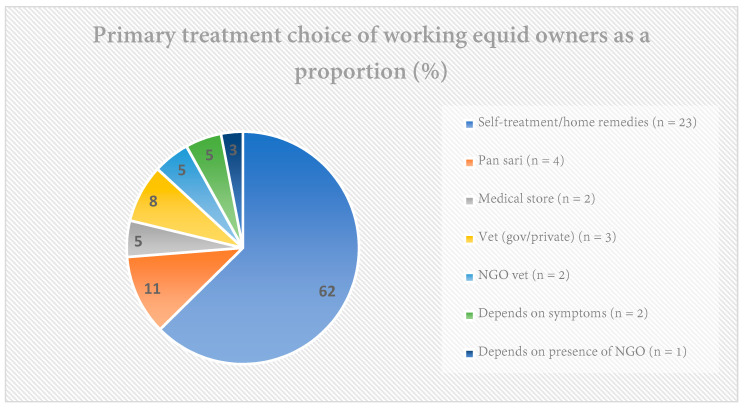
Primary treatment choice of working equid owners as a proportion.

Sixty-two per cent (*n* = 23) of owners relied first upon themselves to treat their animals with home remedies or traditional medicine. Of these, 78 per cent (*n*= 18) would go on to seek help from a doctor (private, government, or NGO-based) if their own treatment did not achieve the desired result. Conversely, five per cent (*n* = 2) of owners stated that they never used home remedies (Figure 2). The majority of those self-treating their animals expressed confidence in their knowledge of their animals and how to treat them. However, few methods of treatment were the same across different animal owners, even within small communities.

The term ‘self-treatment’ in this paper refers to any treatment administered by an owner without prior consultation with a professional healthcare provider. This might include the use of previously bought Ayurvedic or allopathic medicine, as well as the home remedies described below. However, very few respondents referred to the use of allopathic medicine in descriptions of ‘self-treatment’.

For the comparison of health scores between owners who self-treat and those who chose other options, three owners were excluded due to ‘uncertain’ treatment choice. Equid health was not significantly different for owners whose primary choice of treatment was to self-treat (mean health score = 71, *n* = 21) compared to those who preferred other treatment types (mean = 63, *n* = 8; t = −1.2, df = 27, *p* = 0.24, md = 8, 95%, CI = −5.5 to 21). Although one owner, whose primary treatment choice was an NGO vet, had a higher health score than those who self-treated, excluding this owner from the analysis did not affect the statistical results. On average, owners who chose to self-treat owned 1 equid (range = 1 to 4), whilst those who chose other options owned 6 equids (1 to 21).

**Figure 3:**
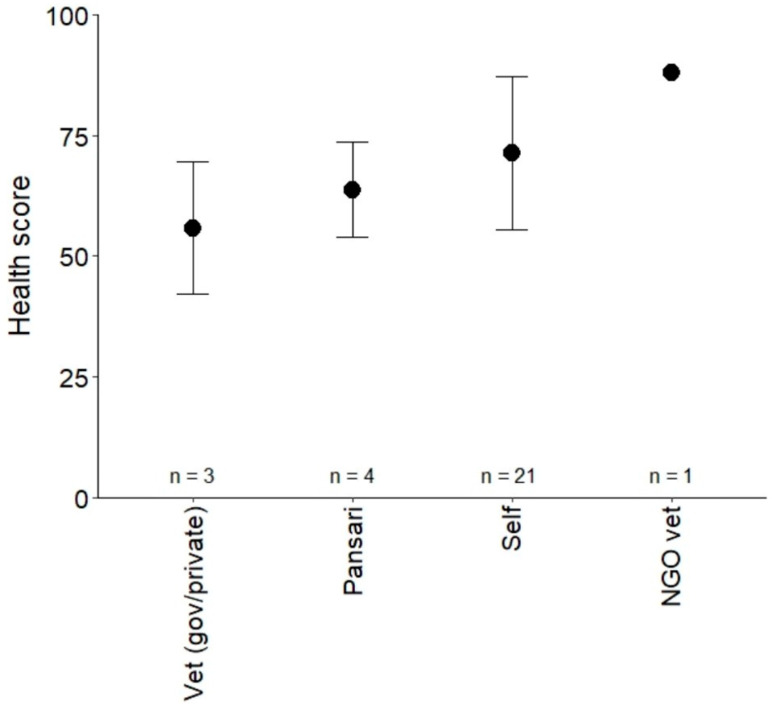
Mean equid health score for equid owners who selected each primary treatment type. The heath score represents welfare in terms of signs of illness, coat health, respiratory health, and skin system alterations, from 0 (worst welfare) to 100 (best welfare). Error bars represent one standard deviation from the mean.

When considering each treatment type separately, the one owner whose primary treatment choice was an NGO veterinarian (*n* = 1) had the best welfare in terms of health, though more owners selecting this option would be needed to verify that this is the norm. Owners whose primary treatment choice was a veterinarian (governmental or private) (*n* = 3) had equids with the poorest welfare, which was, on average, 15 per cent lower in terms of health score than owners who chose to self-treat (Figure 3).

### 3.5. Examples of Self-Treatment

Qualitative data revealed that a variety of measures were used under the ‘self-treatment’ umbrella. 

For example, alcohol in the form of liquor (for human consumption) was given for fever or constipation at quantities of 400–500 mL. In the event of an animal showing signs of a cold or nasal discharge, one respondent described forcing the animal to inhale smoke from a burning jute sack, another forces their equid to inhale smoke from burning grass, while others feed their animals mustard oil (alone or mixed with jaggery—a concentrated cane sugar product) or a concoction of boiled tea leaves with jaggery. Colic was most frequently referred to in terms of self-treatment measures, with 21 different treatment measures described (Table 1).

### 3.6. Influencing Factors for These Decision–Making Practices 

Interviews revealed that the decision-making processes of working equid owners in the study regions related to treating equid disease and injury are affected by four principal factors: infrastructural factors, community characteristics and experiences, owner characteristics and experiences, and economic factors. 

### 3.7. Infrastructural Factors

Traditional medicine or home remedies were rarely the only available source of healthcare in the regions studied, despite the rurality of some of them. Veterinary practitioners, working equid non-governmental organisations, and/or medical stores were prevalent in many of the locations. However, access to, and the availability of, various routes to treatment varied for different participants. While some stated that they had access to numerous routes of treatment from their work site, such as public and private veterinarians, NGO-funded veterinarians, government-run ‘animal hospitals’, and pharmaceutical outlets selling treatment for animals, a minority communicated that access to all routes was limited as some routes to treatment were either unavailable or too far away. Others stated that the limited response rate of veterinarians to brick kiln workers in the local area proved a constraint, suggesting that delays in responding to phone calls ended in fatalities for their animals.

They would try and call someone. Sometimes they don’t have the telephone number. So if they don’t have the phone number, they give a call to somebody, they don’t pick up the call, then they give a call to another number, they don’t pick up the call, and ‘my donkey has a problem in the morning’, and it will be such that by afternoon it would aggravate to such an extent that the donkey dies, so there have been circumstances like that.

(focus group 2 respondent)

Some respondents chose not to follow certain treatment routes due to a perceived lack of knowledge associated with available practitioners in the area.

There is a hospital in the next village but we don’t go there because they don’t know much about animals other than buffalos.

(equid owner)

Several respondents referred to the lack of availability of the required medicine at local medical stores.

First going to the shop. If you don’t get it, to the other shop. If you don’t get it, […] go very far away to buy the medicine”.

(equid owner)

Due to the use of bonded labour in brick kilns [31], it is occasionally the case that owners themselves are not ‘allowed’ to request the attendance of practitioners at the site, instead having to seek the permission of the brick kiln owners. One respondent told of local doctors being unresponsive to direct calls from owners but responsive to calls from kiln owners, disempowering the owner and endangering the health of the animal. By being forced to surrender control to the brick kiln owner, an additional ‘middle-person’ in the chain of help-seeking can add unnecessary delays to treatment.

Sometimes there is a certain amount of nonchalance as well. Because the owner of the kiln will be like, I’ve given some medicine, don’t worry, it will be fine, and maybe the problem the animal is going through is larger than what the [kiln] owner knows. And it blows out of proportion and by the time help comes it’s too late.

(focus group 2 respondent)

Brick kilns in India frequently move their location and, as the majority of equid owners migrate to their places of work from their home villages, this means that it is often the case that each year they are required to work in a location which is new to them. Their knowledge of the local infrastructure is therefore diminished and experience of, and trust in, external practitioners needs to be rebuilt each time.

We have just moved here. We don’t know this area very well. We’ve just started working one month back so we don’t know what facility to find vet, whereas wherever we are back at home, we know doctors, we know places to go in [but] because we change brick kilns very often, we don’t know where to go.

(equid owner)

Although government veterinary hospitals are available in urban, peri-urban, and some rural areas, focus group respondents revealed that they only stock limited medicines. Those medicines are freely available to owners of working equids; however, these respondents reported sometimes having to send animal owners to private pharmacists due to lack of availability of the appropriate medicines. The result of this is that the owner then has to pay for the treatment. If a hospital is not available, local dispensaries tend to take their place. According to registered practitioners in the study regions, most donkey or mule owners would have access to either a hospital or a government dispensary. Despite this, 62 per cent (*n* = 23) still chose to self-treat.

One actor frequently mentioned with regard to help-seeking for treatment was the pansari, who was often referred to in terms of preventative efforts rather than curative medicines, and was used for both humans and animals.

This kind of vendors you’ll find in the equine fairs. He will have a big stall and he will mix those spices and mash it up, and most of the time it does not contain anything allopathic or anything which may cause concern or anything. These are mix of spices, so equine owners, they have a tendency like, if our animals get a dose of spice, maybe once or twice a week, it will never get colic. That has come historically because equines are kept from Ashoka time, in India, so historically we have horses from long time, and there are practitioners which we have inherited from, moguls, from Britishers, from our own country people, so there are many things which has influenced this kind of practices.

(focus group 2 respondent)

Pansari appear to be well-regarded by working equid owners where their services are available. Limited data are available regarding the training requirements or knowledge of pansari in India.

### 3.8. Community Characteristics and Experience

Overall, peer-to-peer advice appeared to be common when seeking information about treating a sick animal, but self-reliance emerged as being of extreme importance to many of the owners. Due to the working model of the Indian brick kiln, and the fact that the majority of the workers—and, by default, their animals—are not normally located at those sites but at home in their villages, brick kiln communities are not necessarily as cohesive as village communities, where everybody knows, firstly, one another and, secondly, where they fit within the hierarchies of knowledge and expertise. To ask for help is to admit a lack of knowledge or experience, which might explain why so many different treatments were administered for the same symptoms, often by respondents living and working in the same brick kilns. Actual home remedies chosen might not be representative of the brick kiln area but of the areas from which the workers originated. At least 26 different towns or villages were referred to as permanent places of residence among the 37 respondents, demonstrating the vast difference in origin among owners.

However, some respondents did refer to ‘gurus’ or other brick kiln workers to whom everybody went to for advice regarding their animal’s illness. Many agreed that peer-to-peer help-seeking was a frequent behaviour.

The owners interact with each other about the problems and who has the experience or who is more experienced.

(equid owner)

Some owners seek advice from local animal owners, even across genera (such as from owners of buffalo), and place confidence in their local plant knowledge to treat their equid. One owner, when queried as to what their animal was being treated with under these circumstances, replied, ‘I don’t know, people go pluck it and bring it. They don’t tell us what it is’.

Assemblages of knowledge are created using what some described as ‘ancestral knowledge’ combined with the input of the immediate community in situ, causing the constant re-creation of treatments and practices as equid owners move around and between kilns.

Yes, there is culture and practice. People do believe in the culture and practice. They follow the same thing what their over and over generation actually doing.

(focus group 1 respondent)

### 3.9. Owner Characteristics and Experience

Often the reason for choosing to treat an animal with home remedies first was not due to the cost of allopathic medicine but due to the belief that home remedies are just as, if not more, efficacious than allopathic medicine. This might be explained by the history of traditional medicine in India and it being an integral aspect of everyday life all over the country. This is demonstrated by the fact that, often, an NGO presence exists where free treatment and allopathic medicine is available and yet many animal owners still choose to self-treat before calling the NGO veterinarians.

The homemade medicines work better on these animals than anything else.

(equid owner)

Those who preferred to self-treat occasionally referred to poor past experiences with animal health practitioners, causing a loss of faith in allopathic medicine while at the same time encouraging a family or community to also shun such treatment.

There was a mule in this area, and it had some issue, so there was a doctor which was called from Rewari and he gave nine injections in the neck. So the neck really swelled up. He was working as a construction worker then, a labourer then. He had come back, the people in the village told him that this has happened. He mixed masalas and gave it to the mule and it worked better.

(equid owner)

Such limited circumstantial evidence can nevertheless create new associations with ideas of treatment efficacy [26] regardless of its *actual* efficacy.

People put phenyl in the wounds […]. Once they put phenyl, the insects die. Like, the parasites die. But if the cut turns into a burn, […] we are asking more about why do they do it. So he says it’s because you see your peers do it. For somebody it has worked, so I’ve got into a practice. Say I have an animal which has a wound, I have applied a little bit of phenyl and it gets better, and then I start trusting in that, and the word of mouth passes and others pick up the same habit, and it just goes around because it’s cheap. Like, it’s easy to do. Convenient.

(focus group 2 respondent)

Convenience emerged as another reason for equid owners to self-treat their animals, relating to time, transport, or financial limitations.

So he asks them, why didn’t you call us, like you could have gone to the doctor. He’s like, this works, so I’ll just use this. It’s convenience

(focus group 2 respondent)

Attitudes towards the use of professional sector practitioners varied considerably. For a number of respondents, a sense of pride existed in declarations that they had never needed to take their animal to an allopathic doctor. It was clear that they wanted to demonstrate their ability to take care of their animals without the need for outside intervention.

I’ve had seven to eight mules before this and I’ve never had the need to give allopathic medicine to my mules.

(equid owner)

It’s a very old animal yet I’ve never had the need to give allopathic medicine.

(equid owner)

Although some may not have been entirely honest about these interactions, focus group respondents corroborated the fact that many people do try traditional methods before calling a doctor. Even where free NGO assistance was available, many still preferred to try their own home remedies prior to calling one of the NGO vets.

The traditional medicine works really well and even when they come […] to put a camp here, that is every six months, we don’t take our mules there. There is no point taking it there.

(equid owner)

This tended to be the case for those with little experience of working with NGOs, as some owners who had spent several years working in NGO-heavy intervention areas showed higher levels of awareness and knowledge regarding animal health management. One respondent reported having abandoned the use of traditional medicines completely in preference of allopathic medicine due to the continued presence of an equid welfare NGO and education gained via various programmes. However, it was also the case that owners who were aware of the presence of NGO services did not always access them.

Many owners referred to the resilience of their mule or donkey, suggesting that, due to this resilience, the animals do not need treatment or do not, in fact, get sick. One owner stated how a deep wound would not need treatment or stitches because ‘their body is such that it heals in two or three days’ (Equid owner). Young animals specifically were described as ‘able to cope’ with pain. Another owner described a mule suffering from severe stomach issues and, rather than seek help from a doctor, he gave the animal away. Eighteen respondents revealed their hierarchies of treatment with regard to their equids and other animals owned. Only six of these respondents stated that they would choose to treat a mule or donkey before another animal, the reason offered in these instances being that the mule or donkey was of more value than a buffalo or goat. Overall, horses were more likely to receive treatment before a mule or donkey, as ‘a mule is much stronger’ (equid owner).

I would first treat the horse, because the horse is a more sensitive animal.

(equid owner)

Seven respondents related the health of their animal to influences of a cosmic nature, believing that the destiny of an animal is already determined by fate or a god. Such beliefs also influence decisions around managing the health of an animal, with some choosing to let god decide as opposed to intervening themselves.

It’s all god’s mercy. If the mercy is there, the donkey will get treated. If there is not, he casts an evil sight over the donkey then it will die anyways […] Everything is done by god.

(equid owner)

Even some qualified veterinary practitioners attributed certain illnesses, diseases, or animal deaths to ‘luck’.

It’s the luck. If the donkey has to die, it will die. If the donkey has to live, it will live.

(focus group 1 respondent)

It is important to bear in mind that further constraints exist in the process of seeking information regarding health management for equid owners working in brick kilns. Most of the owners are illiterate, so accessing written information, such as in books or pamphlets, is often not available to them. Computer literacy and access to the internet is also extremely uncommon among many of the kiln workers.

### 3.10. Economic Factors

Income paid by brick kiln owners can influence the decision-making processes of donkey/mule owners, although many said that money was not an issue, as kiln owners or peers were willing to lend if necessary.

The donkey is helping them earn 50,000 rupees, 1000 rupees is a very small proportion of what the donkey is giving me, so they will think it is an investment […] If they need money, they will borrow it from the brick kiln owner or other persons in the community.

(focus group 1 respondent)

While economic factors did not appear to rank highly overall at the initial decision-making stage, the majority of equid owners, when queried regarding costs of private veterinarians, stated that they were too expensive, referring to either consultation fees or the price of treatment. This also applied to the cost of medicine bought directly from the local pharmacy. However, due to the prevalence of government animal hospitals and veterinarians, even in the rural areas visited, as well as the presence of equine welfare NGOs, for those who did at some point require the services of a veterinarian, costs were reported as being low overall. Several equid owners stated that costs would rise when outside of the specified brick kiln regions, such as at a home town or village, due to the decreased accessibility of government hospitals or NGOs. These costs might contribute to the prevalence of self-treatment as the primary treatment choice for animal owners, although there was no direct evidence to support this.

Only 34 per cent of all respondents reported owning their own means of transport (in all cases a motorbike), the lack of which further impeded the ability of the remaining 66 per cent to access some of the professional care available.

Where finances were reported as a constraint on seeking the assistance of animal health practitioners, the presence of an equid welfare NGO was cited as an alleviation of difficulty by those who made use of NGO intervention. 

### 3.11. Potential Risks of Certain Treatment Choices for Equids

Although the data are limited, there appears to be no significant difference, in terms of outcome, between those animals self-treated by owners and those treated by animal healthcare practitioners; however, the cohort was very small, so further sampling would be required to confirm this result. This might suggest that the home remedies work or that practitioners are not sufficiently trained in treating equids. Adoption of certain treatments could also be attributed to ‘fluke’ recoveries, whereby an animal administered a particular treatment recovered at the same time as the treatment was administered, but as a coincidence rather than a consequence of the treatment. It is worth noting that owners who chose to self-treat owned had, on average, fewer equids than those who sought external help, although the impact of this difference was not explored further. What became clear from the findings was that risk is involved where knowledge is limited.

Sometimes the traditional medicines they work, and sometimes they do not. Many animals have died.

(equid owner)

While it is important to recognise the importance and power of the ability to self-treat an animal, it must be ensured that such treatment is safe and effective.

There are a lot of people who don’t know what proportions of traditional medicines to give, because they can be overdosing that as well. And if there is somebody who is knowledgeable about it, then good. But there are people who don’t have any knowledge about it and they can […] give an overdose of herbs as well, which is not good for the animals and that has also caused death in animals […] People who are not well trained or have little experience of how to do it with traditional practice can always go wrong.

(equid owner)

A lot of the remedies they use herbs, for example turmeric, or even mustard oil. These things do good, because they are Ayurvedic. There have been a history of us using it, it has worked. But if they do something as extreme as pouring phenyl on it, or hot oil, they [an NGO] would try and warn the people who are practicing such methods that this is actually harmful for your animals.

(focus group 1 respondent)

Some of the methods are really weird and they do the damage. Like they sometime use lubricant oil used in the engines, engine oil. So they use it on the wound and that does the damage. They sometimes put petrol to, you know, kill the external parasites […] Some more harsh practices would be cutting tips of ear in tetanus cases, firing the joints for lameness cases, that’s quite prominent in western UP area […] that’s 60 to 70 per cent people does that, thinking that will heal lameness.

(focus group 2 respondent)

Several focus group respondents referred to a ‘big gap for equine healthcare in India’ for which improved training on behalf of the animal healthcare practitioners themselves was perceived as necessary. This was particularly the case in semi-urban or rural areas.

If [there is] a vet who is trained and competent but he is posted in a city, he won’t be going out 30 km to a village to attend a case. Animal in colic won’t go to the hospital, he’ll probably seek the service at his brick kiln. So it is just not competent people.

(focus group 2 respondent)

At the same time, it was recognised that some health interventions by equid welfare organisations had succeeded in modifying equid owners’ behaviours and beliefs regarding some ‘traditional’ methods. Nasal cutting is a method employed by a number of societies, operating on the belief that it cures a variety of ailments in equids [32].

Nasal cutting. I mean, it is one of the major challenging issues of [name of] project […] Earlier the cases are actually 80 or 90 per cent. If you say 100 donkeys, there are 80 or 90 donkeys number of donkeys having nasal cutting. Now if you are visiting in that same locality now it is just about three or four per cent, a 90 per cent change has happened there. So everyone is educated, everyone is completely aware.

(focus group 2 respondent)

## 4. Discussion

By challenging the assumption that the treatment behaviours of equid owners primarily depend upon economic influence [7,9] or access to veterinary services [11], this paper explores the full combination of variables affecting decision-making and help-seeking behaviour. It also investigates how different decision-making processes might impact the outcome of an animal. This study shows that the most common health issues presented by respondents’ equines loosely reflect the findings of Singh et al. [7] described in Section 1.1. Further findings demonstrate that a large proportion of owners of working equids prefer to self-treat their animals before seeking help or advice from a healthcare practitioner but that this decision is influenced by a combination of infrastructural, community, personal, and/or economic factors, rather than being driven by financial or accessibility circumstances alone. Similarly, a study of working equids in Ethiopia [5] discovered that out of 38 owners of working donkeys in Mekelle City, 10.5 per cent used traditional treatment, 31.6 per cent visited the veterinary clinic, and 57.9 per cent of respondents chose to take no action at all when their animal showed signs of illness. Kumar et al. [5] attribute such neglect to the ‘low status’ accorded to donkeys in the area, despite the fact that donkeys unable to work can have a significant negative impact on the livelihoods of their owners. A similar study by Amante et al. [33] on health and welfare assessments of working equids, also in Ethiopia, found that out of 411 owners, 10.6 per cent treated their animals using traditional methods. This suggests that, overall, the numbers of equid owners preferring a self-treatment or traditional medicine route appear to be higher in India as compared to a country such as Ethiopia. This is likely to reflect the culturally entrenched legitimisation of traditional medicine in India itself as compared to other countries. The commonality between similar studies in Ethiopia and our findings is that animal owners adopt the use of either human or animal faeces or urine and high-energy and cheaply-produced substances, such as honey (in the case of Ethiopia) [34] or jaggery (in the case of India), as well as locally used oils or plants. This suggests that a ‘biophysical factors’ category might also be added to those previously set out, as owners utilise substances and compounds which they have access to easily and which are, in many cases, freely available to them within their locality. 

A further possible reason for preferring to self-treat might include the fact that people used to living in rural areas operate within communities where everything tends to be connected, meaning they are likely to feel more comfortable with that which is familiar as opposed to a service delivery which is less personal [35]. In the case of equid owners, the majority of whom are mobile and work away from home communities, such behaviour might be further exacerbated by the lack of familiarity of service providers operating around regions with dense populations of brick kilns and construction sites. According to Wainer [36], rural people ‘are accustomed to assessing the quality of information/service on the basis of being able to locate the provider in their social context and personal history’ [36] (p. 82). This supports our findings in which confidence in local animal health practitioners was low and owner perceptions linked with bad experiences or associated gossip. The building of trust over a short space of time is difficult, particularly for providers with whom owners have limited interaction. A high frequency of veterinary consultancy was found to promote ‘better veterinary-client relationships’ among animal owners in a study of dairy farmers in northern India [37]. With regard to working equid owners, the advantage lies with practitioners working for NGOs, as their presence tends to be constant, allowing relationships to be formed and trust to be established. While NGO vets might not have been the primary choice of treatment for a sick animal for many of the equid owners in the study, they were often the secondary choice where self-treatment failed, and NGO-heavy areas showed higher levels of trust in NGO-related animal health practitioners among animal owners. 

Ilukor and Birner [38] ascertained that ‘livestock farmers who have no skills or training in veterinary science are not able to measure the quality of services being offered’ [38] (p.1) and, as a result, this inability to assess service quality might negatively impact upon their willingness to pay a fee to see them. This might lead to highly qualified practitioners being forced to accept low prices within certain markets, ultimately nudging them out of such areas altogether. Such a circumstance can result in an occupational vacuum which is ultimately filled with individuals professing to be trained animal health practitioners but who may have no formal (or informal) training at all. Thus, constraints not only include cultural tradition and inertia but also the possible inability to assess which service, including self-treatment, might be the best quality to treat the symptom in question. 

With ‘handed-down’ knowledge being a likely determinant behind treatment decisions, normative beliefs, as described by Ajzen [12], indubitably drive behaviour intentions among working equid owners. However, decision-making and health practices are not entirely dependent upon peers or the communities within which working equid owners operate. While certain actors may play a stronger role than others and thus influence the decision-making processes affecting animal health management, ‘indigenous knowledge, traditions and religion can have much influence’ also [39] (p. 691). The decision to self-treat is therefore somewhat dependent upon indigenous knowledge and traditions in the home village communities of the equid owners rather than those of the regions within which they work, thus explaining why proposed treatments for colic and abdominal disorders varied so extremely among respondents, even those working side by side. At the same time, it appears that much knowledge has been lost and the inconsistent responses amongst donkey and mule owners to various diseases, ailments, and injuries might also suggest an ad hoc approach to self-treatment as opposed to an entrenched cultural system of traditional medicine associated with that genus. Such a claim would require further longitudinal studies to be conducted regarding inherited knowledge and how it manifests, perpetuates, and evolves according to the factors of influence described within this paper.

Wathen and Harris [35] referred to methods such as self-treatment and community peer-to-peer help-seeking as constituting the popular healthcare sector, involving common beliefs of non-specialists developed at individual, family, and community levels. Our findings show a similar hierarchy, where equid owners themselves define the illness and employ their associated behavioural, normative, and control beliefs in making a decision to seek help from within the popular healthcare sector or from a professional. A treatment can be legitimised or invalidated regardless of the sector from which it originates according to its perceived efficacy. Thus, home remedies witnessed to be effective can hold higher value than an allopathic treatment deemed to have zero or a negative effect. The latter is further compounded by animal healthcare practitioners operating illegally with little to no formal training, as well as by poor advice delivered by individuals working in veterinary drug retail outlets [14].

Several owners revealed that seeking help from a veterinarian was often their last option should all self-treatment interventions fail. Evidence exists elsewhere of small farmers in India only seeking veterinary assistance when a disease has reached an advanced stage [37]. Similarly, donkey owners in Ethiopia only presented their animals to veterinary clinics when numerous traditional efforts to cure the animal had failed [5], a pattern replicated in the findings of this study. Studies in the eastern Haryana region of India demonstrated that large dairy farmers were less likely than small farmers to use traditional medicine [37]. While this can be attributed to economic resources and education, veterinary consultancy remained relatively high among small farmers compared with our donkey-owning group. Treating animals differently is due, in part, to the background of each occupational group (religion, caste, etc.); however, our findings also reveal that perceptions concerning the constitution of a mule or donkey contribute to the decision-making process. As our study has shown, a misconception still exists that donkeys and mules do not need treatment, especially in comparison with other species, including horses. According to Burden and Thiemann [40], ‘donkeys display different signs and symptoms of pain and sickness than horses or other species’ [40] (p. 379), which could be alternatively described as subtle. This appears to be a substantial driver of owners ignoring issues, as they genuinely believe that donkeys and mules do not feel pain despite there being ‘no evidence that the donkey has a different pain tolerance to that of other equines’ [40] (p. 379). Limited empirical data exist detailing such misconceptions regarding donkeys and mules elsewhere but the small number of respondents in this study adhering to a similar principle demonstrates a requirement for interventions encouraging behavioural change and knowledge extension to further address this issue. 

Pearson and Krecek [41] suggest that remoteness from veterinary services might require ‘greater emphasis on preventative measures and local remedies’ [41] (p. 93). One possible solution to ensure increased welfare of working equids is the continued intervention services of NGOs such as Brooke, the Donkey Sanctuary, the Society for the Protection of Animals Abroad (SPANA), and other charities that provide fixed and mobile clinical services, as well as education and outreach programmes. In such intervention areas, it has been shown that, where services were made available in the Oromiya National Regional State of Ethiopia, uptake of NGO clinical services increased by 95 per cent and the use of traditional medicine decreased [42]. However, reliance on such clinical services presents a danger in that if those services are later withdrawn for whatever reason, they could potentially leave a dangerous legacy within working equid-owning communities, the members of which may have acquired new beliefs regarding traditional medicine but who are unable to afford or access allopathic medicine, leaving animals completely untreated [2]. Such a phenomenon could be described as ‘treatment paralysis’ and prove hazardous for many equids across the world. Attempts to re-configure some of the belief systems feeding into decisions regarding equid welfare should only be attempted when all influencing factors are identified and understood, so that the intention to perform a behaviour originating from the alignment of behavioural, normative, and control beliefs is not stalled or interrupted. As recognised by Luna and Tadich [43], it is important to examine further the ‘human attributes that influence owner-equine interactions’ [43] (p. 1), such as empathy, attitudes towards animals, perception of pain, and their locus of control, and see how these might also be understood with regard to decision-making in animal treatment. They suggest that, where socio-economic constraints do not impact upon the welfare condition of equids, such as in Lanas et al.’s [44] study in Chile, the ability of the owners to empathise might play a more significant role. 

### Limitations of the Study

The sample size for looking at treatment decisions and animal welfare scores was small, so statistical results should be viewed with caution. Further sampling would be required to draw conclusions with a greater level of certainty, so generalising the data is not possible at this stage. Most owners were unable to name ailments, such as Surra, so pinpointing the exact ailment during the study was, on occasion, difficult; in these cases researchers ascribed the ailment according to symptoms described. There also appeared to be some confusion as to what constitutes a charity or NGO as opposed to regular government vet practitioners. Equally, many animal owners appeared to be unable to differentiate between allopathic (often called “English medicine” in day-to-day language) and Ayurvedic medicine. Ayurvedic was then also sometimes difficult to distinguish from other methods of treatment regarded as ‘traditional’ but which were not Ayurvedic. 

## 5. Conclusions

This study demonstrates that the decision-making processes of equid owners with regard to treating their animals are influenced most strongly by behavioural and normative beliefs and that imposition on their control beliefs impedes their desire or ability to seek help outside of their own, often limited, knowledge. As in the Anthra project in other parts of rural India, ‘as the economic survival of these families is dependent on the livestock they own, validated ethnoveterinary knowledge assumes great importance’ [9] (p. 121). Any attempt to intervene or influence belief systems regarding animal health management must always seek to ‘learn, evaluate and without being biased and ethnocentric, promote and integrate the beneficial facets of traditional animal healthcare practices into primary livestock healthcare delivery services’ [45] (p. 1). Examples might include recruiting well-regarded members of communities to act as goodwill ambassadors to encourage safe home-treatment remedies or increasing interventions among younger members of communities. The network of actors within which the owner of a working equid operates is significant, especially in rural areas, and Wathen and Harris [35], although referring to human health practices, state that key actors exist within those networks who can ‘facilitate or constrain information exchange’ and who should therefore also be taken into account [35] (p. 641).

Healthcare practices cannot be considered as separate from general welfare management of equids. All other owner-, community-, infrastructure-, and economic-related factors must be taken into account in considering health and well-being. In addition to this, accessibility to other animals for social purposes, suitable housing, food and water, and adequate working conditions [8] can contribute to animals’ well-being and prevent unnecessary disease or injury. Further validation should occur of current practices employed in order to identify effective or dangerous practices and behaviour change should be encouraged in order to adopt effective methods. The need exists to implement a ‘best practice, basket of options’ approach [46] which would enable equid owners to safely self-treat their own animals, while at the same time ensuring the awareness of, and accessibility to, other treatment options.

## Figures and Tables

**Table 1:** Examples of self-treatment measures for colic.

Remedy 1	Soda, tobacco, and noshadar (ammonium chloride)
Remedy 2	Boiled tea leaves with jaggery, salt, and oil (oil unspecified)
Remedy 3	Boiled milk with jaggery
Remedy 4	Mustard oil mixed with lime juice and black salt
Remedy 5	Mustard oil, bicarbonate of soda, and human urine
Remedy 6	Boiled tea leaves with salt, bicarbonate of soda, and Pepsi
Remedy 7	Garmunda (Cirullus colocynthis)
Remedy 8	Farki (unidentifiable)
Remedy 9	Junda grass (unidentifiable) brewed in water, black salt, and mustard oil
Remedy 10	Tobacco mixed with boiled tea leaves
Remedy 11	Himalayan batisa with jaggery
Remedy 12	Himalayan batisa and garmunda (Citrullus colocynthis)
Remedy 13	Human urine
Remedy 14	Jaggery, garlic, onion, and tobacco
Remedy 15	Ginger, garlic
Remedy 16	375–400 mL liquor, tea leaves with salt
Remedy 17	Massage stomach with oil (turpentine, alkanet root, or Rahat Rooh oil)
Remedy 18	Brewed tea with salt and sugar, traditional herbs (details unknown to owner)
Remedy 19	Jaggery, tobacco, and lime
Remedy 20	Mixed herbs (details unknown to owner)
Remedy 21	Leaves of Madar tree (Calotropis gigantea) mixed with jaggery

Details of quantity administered unknown, apart from Remedy 16.

## Data Availability

The datasets generated and/or analysed during the current study are not publicly available due to them containing sensitive material, which do not meet the GDPR guidelines.

## Supplementary Materials

Supplementary materials are available online at https://www.mdpi.com/article/10.3390/ani11051307/s1.
