# Supplementary material for: ‘Don’t Put the Cart before the Mule!’ Challenging Assumptions Regarding Health-Related Treatment Practices of Working Equid Owners in Northern India
Source: Animals (Basel). 2021 May 1;11(5):1307. doi: 10.3390/ani11051307 (PMC8147387; doi:10.3390/ani11051307)
Supplement: Supplementary file 1 [file animals-11-01307-s001.zip › animals-1015283-supplementary.pdf]

**Livelihoods Survey**

| | |
|---|---|
| Type of location | Brick Kiln |
| | Village |
| | City |
| | Pharmacy |
| | Other |
| Name of location | |
| Person ID | |
| Do you consent to participating in this survey | |
| Age | Under 18 |
| Thank participant for their time. Please stop the survey. | |
| | 18-30 |
| | 30-50 |
| | Over 50 |
| | Prefer not to answer (over 18) |
| Gender | Male |
| | Female |
| | Other |
| Place of Birth | |
| Who is head of your household? | |
| How many adults (18 and over) in your household? | |
| How many male children (under 18) in your household? | |
| How many female children (under 18) in your household? | |
| What is your main job role currently? | Donkey/mule owner |
| | Donkey/mule handler |
| | Vet |
| | Agrovet |
| | Drug retail outlet worker |
| | Qualified pharmacist |
| | Other (please specify) |
| Do you have other job roles or sources of income? | Yes |
| | No |
| | Unsure |
| | Prefer not to answer |
| What is your personal weekly income (in total, including money saved and spent)? | |
| How much does your whole household earn per week (in total, including money saved and spent)? | |
| What is your education level? | None |
| | Primary |
| | Secondary |
| | University/ college |
| | Other (please specify) |
| What is your ethnic group? | |

| | |
|---|---|
| What is your religion? | Hindu |
| | Buddhist |
| | Muslim |
| | Christian |
| | Jewish |
| | Other (please specify) |
| | |
| Is your present place of residence: | Temporary |
| | Permanent |
| Home ownership | Own |
| | Rent |
| | Stay with family/ Friends |
| | Prefer not to answer |
| | Other (please specify) |
| Where is your permanent home? | |
| Where is your temporary home? | |
| Do you own or rent any land in your permanent home? | Own |
| | Rent |
| | No |
| | Other (please specify) |
| Do you have any of the following in your household? | Electricity |
| | Mobile phone |
| | Motorbike |
| | TV |
| | Computer |
| | Car |
| Do you own any donkeys/mules? | Yes |
| | No |
| How many donkeys/mules do you own? | |
| What percentage of your annual income is dependent on your donkeys / mules? | |
| On average, how much do you pay for a donkey / mule? | |
| Where did you obtain your donkeys/mules? | |
| On average how many years do you keep your donkeys/ mules? | |
| What is the average age of your donkeys/ mules? | |
| How many years have you worked with donkeys/mules? | |
| Do you think working with donkeys/mules changes your social status? | Increases |
| | Decreases |
| | No change |
| | Unsure |
